# Rapid prediction of complex nonlinear dynamics in Kerr resonators using the recurrent neural network

**DOI:** 10.1007/s12200-025-00164-4

**Published:** 2025-09-25

**Authors:** Tianye Huang, Lin Chen, Mingkong Lu, Jianxing Pan, Chaoyu Xu, Pei Wang, Perry Ping Shum

**Affiliations:** 1 School of Mechanical Engineering and Electronic Information, China University of Geosciences (Wuhan), Wuhan, 430074 China; 2 Wuhan National Laboratory for Optoelectronics, Wuhan, 430074 China; 3 The State Key Laboratory of Photonics and Communications, Shanghai, 200000 China; 4 Shenzhen Research Institute of China University of Geosciences, Shenzhen, 518057 China; 5 Yazheng Technology Group Co., Ltd, Wuhan, 430200 China; 6 Wuhan Huaray Precision Laser Co., Ltd, Wuhan, 430223 China; 7 Department of Electronic and Electrical Engineering, Southern University of Science and Technology, Shenzhen, 518055 China

**Keywords:** Kerr ring resonators, Cavity soliton (CS), Recurrent neural network (RNN), Nonlinear, Dynamics

## Abstract

Kerr resonator is one of the most popular platforms to produce optical frequency comb and temporal cavity soliton. As an essential method for investigating the nonlinear dynamics of Kerr resonators, traditional numerical simulations rely on solving the Lugiato–Lefever equation (LLE) using the split-step Fourier method (SSFM), which is computationally intensive and time-consuming. To address this challenge, this study proposes a recurrent neural network model with prior information feedback, enabling efficient and accurate prediction of soliton dynamics in Kerr resonator. With the acceleration of graphics processing unit (GPU), the computational efficiency improved by 20 times. We compared various recurrent neural networks and found that the gated recurrent unit (GRU) network demonstrated superior performance in this task. This work highlights the potential of artificial intelligence (AI) for modeling nonlinear optical dynamics in Kerr resonator, paving the way for designing optical frequency comb and generating ultrafast pulse.

**Graphical abstract:**

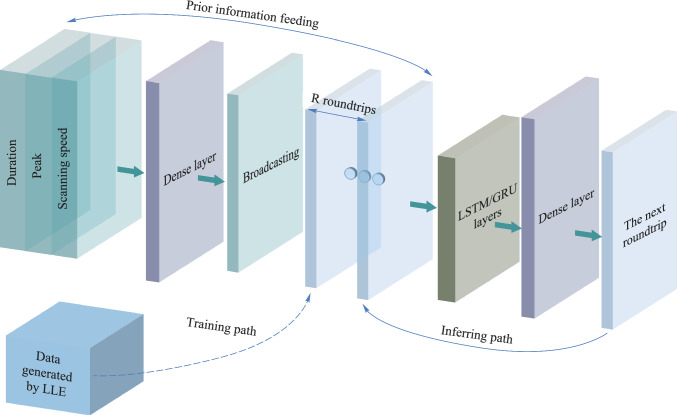

## Introduction

Temporal cavity solitons (CSs) are self-sustained, localized light pulses that circulate infinitely within an externally driven Kerr resonator. Benefiting from the composite balances between cavity dispersion and Kerr nonlinearity, as well as the parametric gain and loss, temporal CSs can maintain their shape during the propagation [1]. The advantage of chip-scale integration endows CS with significant potential for applications in optical memories and all-optical system [2]. Therefore, temporal CSs have garnered significant attention since their discovery [3–10]. The description of CS dynamics is usually based on Lugiato–Lefever equation (LLE) [11–15], which provides a simplified description of nonlinear and dispersive effects in optical systems, effectively capturing the dynamic behavior in nonlinear ring resonators. The split-step Fourier method (SSFM) is one of the primary methods for solving the LLE. However, this method usually requires extensive computational work, and inefficient computing approaches undoubtedly hinder the study and research of temporal CSs.

With the rapid development of artificial intelligence (AI) in recent years, the combination of AI and optics has attracted significant attentions [16]. Deep learning technology possesses unique advantages and is poised to address challenges posed by traditional mathematical models, thereby leading to innovative optical communication models [17, 18]. AI is also applied in the design of optical devices, such as the inverse design of dispersive optical devices [19, 20], automatic optimization of enhanced model-locked lasers [21–24], and performance improvement of optical fiber sensors [25, 26]. The development of graphics processing unit (GPU) parallel computing has greatly improved the computational efficiency of feedforward neural networks (FNN). Most of the research is focused on the application of machine learning to the study of nonlinear dynamics in optics. Currently, AI through FNN can successfully predict ultrafast nonlinear dynamics in optical fibers, with significantly high computational efficiency [27, 28]. In the field of ultrafast lasers, recurrent neural networks (RNNs) can also rapidly and accurately predict the dynamics of femtosecond mode-locked lasers [29].

Utilizing machine learning to predict the dynamics of Kerr resonators poses several challenges. Firstly, the nonlinear dynamics in ring cavities involve process at multiple different time scales, undoubtedly increasing the complexity of analysis. Secondly, complex phenomena such as chaotic state existed in nonlinear ring cavities. Strong randomness makes it difficult for AI to learn and predict. Currently, applying machine learning to the study of dynamics in nonlinear ring cavities remains an unresolved issue.

In this paper, we use an RNN network to model the nonlinear dynamics within a Kerr fiber ring resonator. The model can accurately predict the generation of temporal CSs and other dynamic processes, and the AI model is 20-fold faster than traditional LLE model. This work employs a prior information method, enabling the model to make accurate judgments within Kerr ring cavities under different parameters. Different RNNs computational loads and mean square errors are compared to selecting the optimal solution. It is shown that this approach can significantly facilitate the design of resonators for the generation of CSs.

## Single pump driving

### Data generation using the LLE under single-pulse driving

**Fig. 1:**
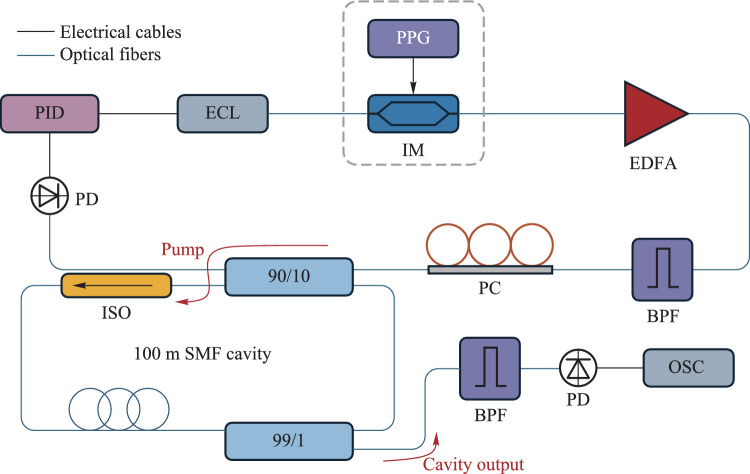
Simulation setup schematic of the Kerr ring cavity under single-pulse driving

Figure 1 shows the simulation model which is based on a fiber Kerr resonator constructed from 100 m of single-mode fiber, closed by a 90/10 coupler. The pulse-pattern generator (PPG) is to generate high-precision pulse signals that provide timing-driven Gaussian pulses. These pulses are subsequently used to modulate the continuous-wave (CW) light source via an amplitude modulator (AM), resulting in the output of modulated optical pulses from the light source. An optical isolator is employed to suppress stimulated Brillouin scattering, while a 99/1 tap coupler is used to enable monitoring of the intracavity dynamics [30].

The evolution of the optical field *E*(*t*, *τ*) within a Kerr ring cavity is represented by the following dimensionless mean-field LLE described by Eq. (1) [11]. In this simulation, we consider a Kerr ring resonator driven by Gaussian optical pulse. The driving field is assumed to be synchronized with the round-trip time of the cavity, exhibiting anomalous dispersion within the resonator. On the right-hand side of Eq. (1), each term represents cavity losses, Kerr nonlinearity, cavity phase detuning, group velocity dispersion, and (rapid) time-varying coherent driving, respectively.

1 $$\frac{{\partial^{2} E\left( {t,\left. \tau \right)} \right.}}{\partial t} = \left[ { - 1 + {\text{i}}\left( {\left| {E\left| {^{2} - \Delta} \right.} \right.} \right) + {\text{i}}\frac{{\partial^{2} }}{{\partial \tau^{2} }}} \right]E + S(\tau ),$$

where the Gaussian pulse *S*(*τ*) driving field is represented by the following:

2 $$S(\tau ) = S_{0} \exp \left( { - \frac{{\tau^{2} }}{{2\tau_{{\text{G}}}^{{2}} }}} \right).$$

Here, *τ*_G_ is the pump pulse width. *S*_0_ is the amplitude of the Gaussian pulse driving field. Δ_s_ represents that scanning the detuning through the cavity resonance. *τ*_G_, *S*_0_ and Δ_s_ are three adjustable parameters, for generating different temporal evolutions of the optical field within the Kerr ring cavity. The pump pulse width *τ*_G_ ranges from 15 to 21 with a step size of 1. The amplitude *S*_0_ of the Gaussian pulse driving field is set from 1.5 to 2.1 with a step size of 0.1. Δ_s_ ranges from 0.004 to 0.016 and a step size of 0.002 each round-trip. When the pump pulse width exceeds the specified range, it leads to an unstable condition inside the cavity, resulting in the chaotic state. To save memory and analyze the soliton formation process in detail, the scanning speed is set within this range. The maximum number of round trips of 1500 ensures a stable state. A total of 343 data sets are generated, from which 200 data sets are randomly selected as the training set. Among the remaining 143 data sets, 70 are chosen as the test set.

**Fig. 2:**
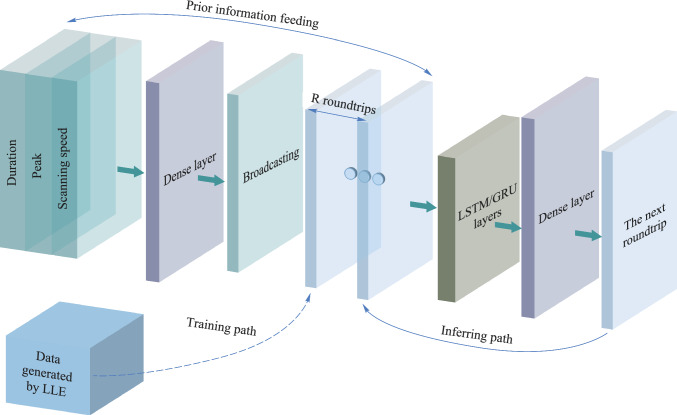
AI model based on prior information

Figure 2 shows the workflow of the AI model based on prior information. The segmented two-dimensional arrays are initially passed through a dense layer. Using broadcasting mechanisms, the 3-column parameter vectors expend to 512 columns, constructing a RNN with sampling points. Then, the data is input into the gated recurrent unit (GRU) layer. Here, two GRU layers are selected with a sequence length of 12. The 12th time sequence is predicted using the previous 11-time sequences. Subsequently, the predicted sequence is utilized as the next input, establishing a sliding window prediction mode.

To evaluate the accuracy of AI modeling, the root mean square error is set as the standard, shown as Eq. (3). Here, *n* is the number of sampling points, *D*_pred_ is the predicted field intensity, and *D*_LLE_ is the result obtained from the LLE. This formula calculates the RMSE for each round trip. To get the overall RMSE, averaging the errors across all round trips and samples is used.

3 $${\text{RMSE}} = \sqrt {\frac{1}{n}\sum\limits_{i = 1}^{n} {(D_{{{\text{pred}}}} - D_{{{\text{LLE}}}} )^{2} } } .$$

### Prediction of temporal CSs

**Fig. 3:**
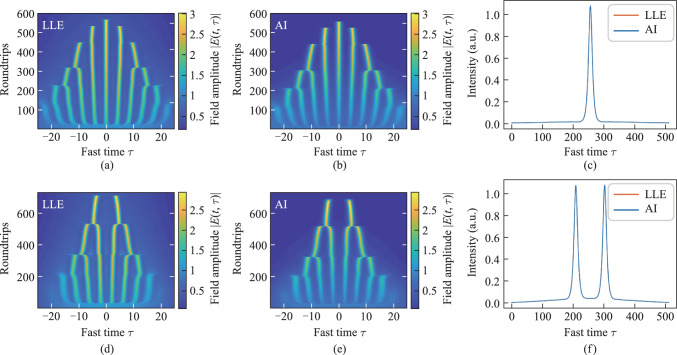
Evolution of the single soliton obtained by **a** LLE and **b** AI (*τ*_G_ = 20, *S*_0_ = 1.9, Δ_s_ = 0.008). **c** Prediction of the state of single soliton formation. The evolution of the dual soliton obtained by **d** LLE and **e** AI (*τ*_G_ = 15, *S*_0_ = 1.9, Δ_s_ = 0.006). **f** Prediction of the state of dual soliton formation

Figure 3(a) shows the evolution process of a single soliton in the Kerr ring cavity as predicted by the LLE, while Fig. 3(b) shows the same process predicted by the AI model. Figure 3(c) compares the intensity during the formation of the single soliton, as predicted by the LLE and AI model. The results demonstrate the AI model successfully predicts the formation of the single soliton. For Gaussian pumping scheme, another possible CSs state is dual solitons. Figures 3(d) and 3(e) show the evolution process of dual solitons in the Kerr ring cavity as predicted by LLE and AI. Figure 3(f) compares the intensity during the formation of the dual solitons, as predicted by the LLE and AI model. The results demonstrate the AI model successfully predicts the formation of dual solitons.

### Prediction of turing ring

**Fig. 4:**
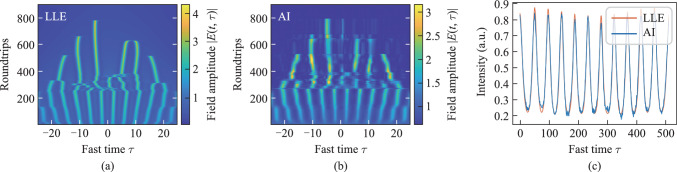
Evolution of Turing ring obtained by **a** LLE and **b** AI (*τ*_G_ = 23, *S*_0_ = 2.3, Δ_s_ = 0.008). **c** Predicted comparison at the 200th roundtrip

When the pump pulse width *τ*_G_ and amplitude of the driving field *S*_0_ increase, turing ring emerge during the initial stage of intracavity optical field evolution. Figure 4(a) shows the evolution process of the intracavity optical field, transitioning from Turing rings to a chaotic state and eventually to a single soliton. As shown in Fig. 4(b), the AI model accurately predicts the formation of Turing rings and their transition into a chaotic state. Due to the random nature of the chaotic state, the AI model cannot fully predict the dynamics within the regime. Nevertheless, the evolution trend matches well.

## Dual pump driving

### Data generation using the LLE under bichromatic driving

There exist significant differences between conventional CSs and parametrically driven cavity solitons (PDCS) in terms of their physical mechanisms and application characteristics. CSs are driven by a monochromatic continuous-wave field and extract energy from the background through four-wave mixing (FWM), with their spectral center coinciding with the driving frequency. In contrast, PDCS rely on a bichromatic driving field and are formed via a phase-sensitive amplification mechanism based on non-degenerate FWM [31]. Their spectral center is located between the two driving frequencies, and they exhibit two opposite phase-stable states. This fundamental distinction endows PDCS with enhanced noise resistance and superior phase controllability. Therefore, investigating the nonlinear dynamics in Kerr resonators under bichromatic driving is of great significance for the generation and control of PDCS.

**Fig. 5:**
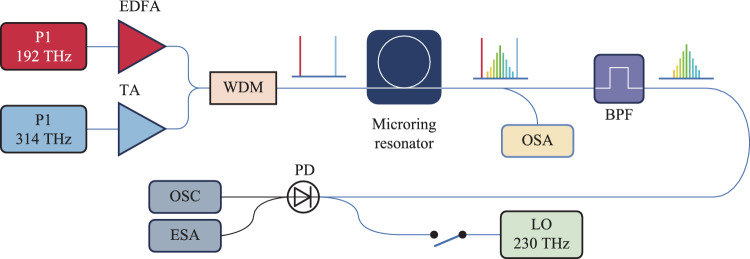
Simulation setup schematic of the Kerr ring cavity under bichromatic driving

The simulated Kerr resonator depicted in Fig. 5 is as follows. One laser can be tuned in the telecommunications C-band, covering a frequency range from 186 to 198 THz (corresponding to a wavelength range of 1515 nm–1613 nm), while another laser can be tuned over a higher frequency range, from 306 to 330 THz (corresponding to a wavelength range of 910 nm–980 nm). Prior to coupling into the resonator through a wavelength division multiplexer (WDM), optical amplification and combination are performed using the WDM to ensure effective coupling at all relevant frequencies. At the output of the resonator, 90% of the signal is directed to a spectrum analyzer for analysis. The remaining 10% is passed through a bandpass filter to remove spectral components around the driving frequency, thereby allowing for the characterization of the signal field generated by parametrically induced effects.

The driving field of the Kerr resonator consists of two coherent driving fields with frequencies of *ω*_±._ During the m roundtrip inside the resonator, the evolution of the evolution of the electric field envelope is governed by the non-linear Schrodinger equation (NLSE), as shown in Eq. (4).

4 $$\frac{{\partial E^{(m)} (z,\tau )}}{\partial z} = {\text{i}}\hat{\beta }_{{\text{S}}} \left( {{\text{i}}\frac{\partial }{\partial \tau }} \right)E^{(m)} + {\text{i}}\gamma |E^{(m)} |E^{(m)} .$$

Here, *z* is the coordinate along the waveguide of the resonator, *τ* is the time, and *γ* is the nonlinear coefficient. The Ikeda map is used to describe the coupling equation between light and the resonator, considering the bichromatic driving. The boundary equation is as follows.

5 $$E_{{}}^{m + 1} (0,\tau ) = \sqrt \theta E_{{{\text{in}}}} + \sqrt {1 - \theta } E_{{}}^{m} (L,\tau ){\text{e}}^{{ - {\text{i}}\delta }} .$$

To realize PDCS in Kerr ring resonator, the simulation assumes a critically coupled resonator (*α* = *θ*), characterized by a cavity length of approximately *L* ≈ 8.3 mm. A nonlinear coefficient *γ* = 1.2 W^−1^⋅km^−1^ and a fitness *F* = π/*α* = 5000. Specifically, the simulation considers both second and fourth-order dispersion, where *D*_2_ = 2π × 4.1 kHz and *D*_4_ = − 2π × 33 mHz. These parameters lead to a pump frequency shift of Ω_p_ = 2π × 30.4 THz, ensuring *D*_int_(*p*) + *D*_int_(− *p*) ≈ 0 for the relative mode number *p* = 1217. For simplicity, it is assumed that the two driving fields are resonant within their respective linear resonators, and each field is a continuous-wave source with a power of 200 mW [30]. To explore the effects of varying system parameters on PDCS generation, the simulation investigated a range of pump power and detuning values. Specifically, the pump power was varied from 0.10 W to 0.20 W in increments of 0.1 W, while the detuning was varied from 0.5 rad to 1.6 rad in increments of 0.1 rad. This parameter sweep allowed for a comprehensive investigation of the system’s nonlinear dynamics across both stable and near-critical regimes. In total, 132 data sets were generated, designed to be both statistically representative and randomized, minimizing the risk of overfitting. The data was partitioned as follows: a training set of 100 sets were randomly selected as the training set for AI model development, 16 sets served as the validation set for hyperparameter tuning, and 16 sets were used as the test set to evaluate the model’s generalization ability.

**Fig. 6:**
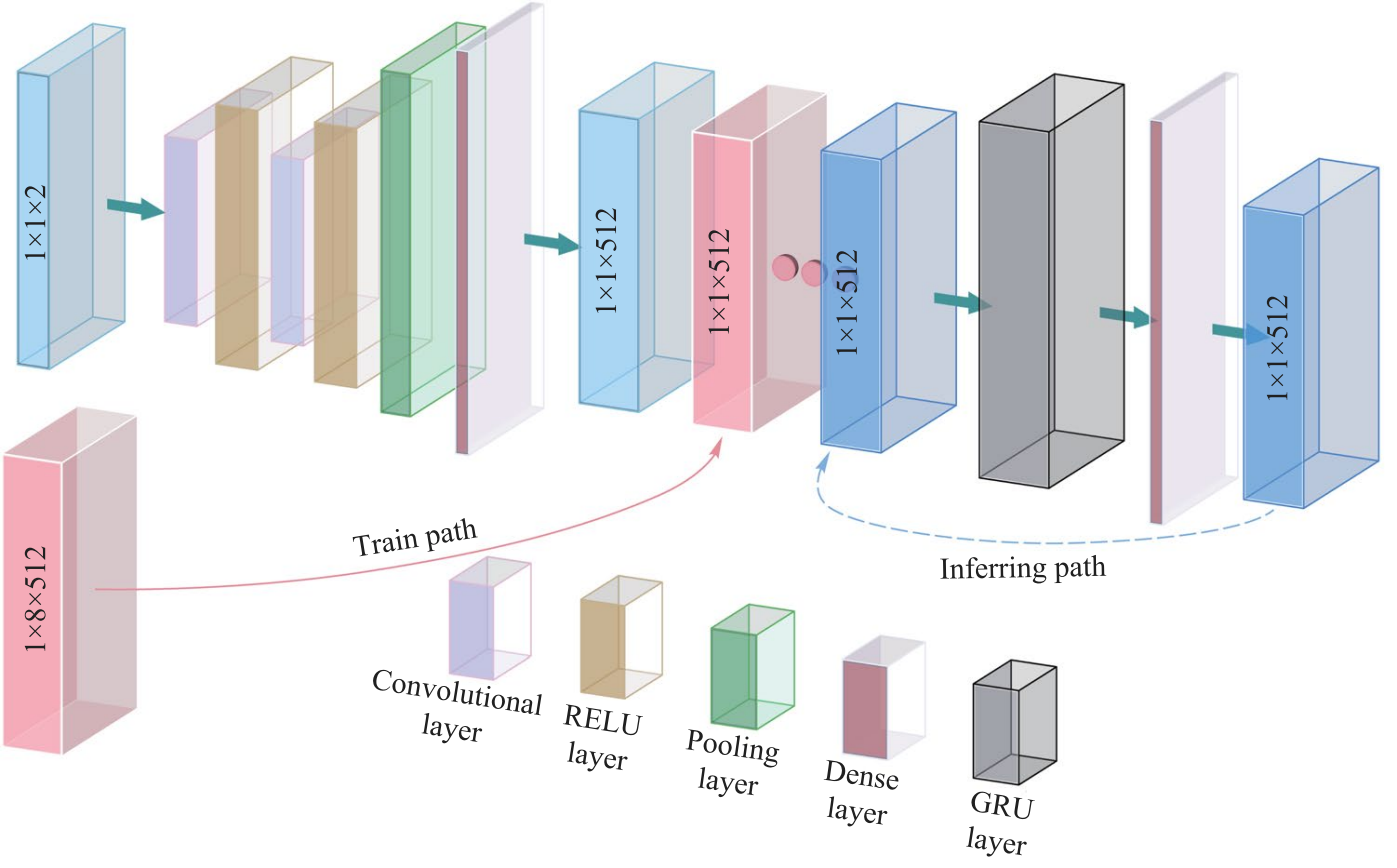
AI model based on the combination of CNN and GRU

Figure 6 shows the workflow of the AI model based on the integration of convolutional neural networks (CNNs) and GRU. The detuning and pump power are processed through CNN to extract representative features. CNN constitutes a feed-forward, hierarchically layered architecture wherein each stratum employs a set of learnable convolutional kernels to effect a cascade of transformations [32]. The convolution operation helps to extract useful features from locally related data points. The output of the convolution kernel is then fed into a nonlinear processing unit, which not only helps to learn abstract features but also embeds nonlinear characteristics in the feature space [33]. The GRU is then employed to handle the temporal sequence data, capturing long-term dependencies. This hybrid approach not only enables efficient processing of complex input data but also enhances the model’s predictive performance and generalization ability in time series tasks. By leveraging this model, it becomes possible to better adapt to variations in temporal data and improve performance across different tasks. After comparative analysis, a sequence length of 8 and 2 GRU layers were selected, which yielded favorable results.

### Prediction of PDCS

**Fig. 7:**
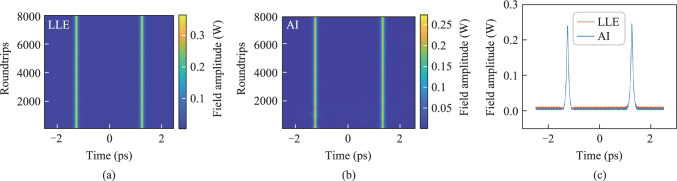
Evolution of the PDCS by **a** LLE and **b** AI (*S* = 0.2,Δ = 1.2). **c** Comparison at the 6500th roundtrip

The formation of PDCS under bichromatic driving conditions requires the fulfillment of three critical criteria. Firstly, the resonator must exhibit anomalous dispersion at the soliton frequency to support soliton formation. Secondly, the effective detuning between the degenerate FWM frequency and the nearest cavity resonance must be sufficiently small to ensure approximate linear phase matching. Finally, the intracavity field at the driving frequencies should remain temporally uniform and stable, providing a constant parametric driving strength. By appropriately tuning the pump power and detuning parameters, the generation and control of PDCS can be effectively achieved. When the pump power is set to 0.2 W and the detuning is 1.2, the intracavity field satisfies the above three conditions, leading to the formation of PDCS within the resonator as shown in Fig. 7. Figure 7(c) show the predictions of the optical field intensity inside the resonator at the 6500th roundtrip, comparing the results from the AI model and LLE model during the generation of stable PDCS.

## Discussion

### Comparison of different network models

To compare the RMSE and computational efficiency of the current AI model with different network models, Table 1 shows the results under single-pulse driving. With similar RMSE, GRU offers faster training time and shorter running time. Regardless of the network model, both LSTM and GRU are one order of magnitude faster than the traditional LLE. Even without GPU, the AI model is still faster. It is worth nothing that GPU acceleration is limited for the SSFM method due to the strong temporal step dependency inherent in its computation, as each step relies on the results of the previous one. This limits the ability to fully leverage the parallel processing capabilities of GPUs, which is more suitable for handling large-scale independent tasks.

**Table 1:** Comparison of different models under single-pump driving

Models	LSTM	GRU	LLE
NRMSE^a^	0.46515	0.46681	N/A
FLOPs^b^	3.07 × 10^11^	2.31 × 10^11^	N/A
Number of parameters	1.52 × 10^7^	1.161 × 10^7^	N/A
Simulation time with GPU^c^	1.82 s	1.63 s	181.60 s
Simulation time^d^	23.17 s	17.14 s	39.69 s

^a^Normalized root mean square error (NRMSE)

^b^Floating point operations per second (FLOPs)

^c^The mean time of 100 simulations over GeForce RTX 2080Ti

^d^The mean time of 100 simulations over Intel^®^ Xeon^®^ CPU E5-2680v3@4.20 GHz

Table 2 compares the traditional GRU neural network with the CNN-GRU neural network proposed in this study. The results show that, with only a slight increase in parameters, the CNN-GRU network achieves a significantly lower RMSE than the GRU, demonstrating better prediction accuracy and stronger learning ability for prior information. Additionally, CNN-GRU outperforms GRU in computational efficiency, requiring only 3.49 s on GPU, nearly 20 times faster than the traditional model. Even without GPU acceleration, CNN-GRU remains almost six times faster than the traditional model. However, without GPU support, GRU is more efficient than CNN-GRU due to the computational demands of CNN layers. Overall, the CNN-GRU model offers a good balance of high accuracy and enhanced computational efficiency, making it an ideal choice for nonlinear optical modeling.

**Table 2:** Comparison of different models under bichromatic driving

Models	GRU	CNN-GRU	LLE
NRMSE	3.53527	0.24473	N/A
FLOPs	7.10 × 10^11^	7.10 × 10^11^	N/A
Number of parameters	1.15 × 10^7^	1.161 × 10^7^	N/A
Simulation time with GPU^a^	7.48 s	3.49 s	N/A
Simulation time^b^	23.24 s	27.293 s	68.12 s

^a^The mean time of 100 simulations over GeForce RTX 4090

^b^The mean time of 100 simulations over 12th Gen Intel^®^ Core™i7-12700H

### Comparison of different GRU layers

In the proposed model, different number of GRU layers yields different RMSE and computational efficiencies. To explore better neural network performance, Tables 3 and 4 compare the RMSE, computational load, and time for different numbers of GRU layers. It can be observed that, through a comprehensive comparison, the two-layer GRU have a good balance between RMSE and computation time. Hence, chose two GRU layers for our network model.

**Table 3:** Comparison of different GRU layers under single-pulse driving

Layers	1 GRU	2 GRU	3 GRU
NRMSE	0.86	0.72	0.67
FLOPs	9.93 × 10^10^	2.31 × 10^11^	3.62 × 10^11^
Number of parameters	5.25 × 10^6^	1.16 × 10^7^	1.78 × 10^7^
Simulation time with GPU^a^	1.49 s	2.1 s	2.76 s
Simulation time^b^	16.82 s	17.14 s	55.24 s

^a^The mean time of 100 simulations over GeForce RTX 2080Ti

^b^The mean time of 100 simulations over Intel^®^ Xeon^®^ CPU E5-2680v3@2.50 GHz

**Table 4:** Comparison of different GRU layers under bichromatic driving

Layers	1 GRU	2 GRU	3 GRU
NRMSE	6.07	0.24	6.84
FLOPs	3.07 × 10^10^	7.10 × 10^11^	3.62 × 10^11^
Number of parameters	5.27 × 10^6^	1.16 × 10^7^	1.78 × 10^7^
Simulation time with GPU^a^	3.02 s	3.49 s	3.89 s
Simulation time^b^	15.01 s	27.29 s	44.40 s

^a^The mean time of 100 simulations over GeForce RTX 4090

^b^The mean time of 100 simulations over 12th Gen Intel^®^ Core™i7-12700H

### Comparison of different time sequence lengths

Different time sequence length affects the model’s computational efficiency and accuracy. To explore better neural network performance, Tables 5 and 6 compare RMSE, computational, and time for different sequence lengths under single-pulse driving and bichromatic driving. Results show that longer sequences increase computation time without GPU cores. Therefore, shorter sequences are recommended without GPU cores, while longer sequences can be chosen with GPU cores for better RMSE. We chose the sequence length of 12, the comprehensive performance is better.

**Table 5:** Comparison of different time sequence length under single-pulse driving

Length	12	14	16	18
NRMSE	0.78	0.78	0.79	0.74
FLOPs	1.98 × 10^11^	2.31 × 10^11^	2.63 × 10^11^	2.95 × 10^11^
Number of parameters	5.25 × 10^6^	1.16 × 10^7^	1.78 × 10^7^	1.16 × 10^7^
Simulation time with GPU^a^	1.49 s	2.1 s	2.76 s	3.04 s
Simulation time^b^	16.82 s	17.14 s	55.24 s	57.33 s

^a^The mean time of 100 simulations over GeForce RTX 2080Ti

^b^The mean time of 100 simulations over Intel^®^ Xeon^®^ CPU E5-2680v3@2.50 GHz

**Table 6:** Comparison of different time sequence length under bichromatic driving

Length	6	8	10
NRMSE	2.45	0.24	0.22
FLOPs	5.34 × 10^11^	7.10 × 10^11^	8.86 × 10^11^
Number of parameters	1.16 × 10^6^	1.16 × 10^7^	1.16 × 10^7^
Simulation time with GPU^a^	1.49 s	2.1 s	3.04 s
Simulation time^b^	16.82 s	17.14 s	57.33 s

^a^The mean time of 100 simulations over GeForce RTX 4090

^b^The mean time of 100 simulations over 12th Gen Intel^®^ Core™i7-12700H

## Conclusion

In conclusion, we provide a novel modeling method for Kerr nonlinear ring cavities. This method, supported by data generated from the LLE, successfully predicts the formation of temporal CSs. With GPU cores, our computation speed increased nearly 20-fold. Even without GPU cores, this method is faster than traditional computational approaches. Single solitons, double solitons, Turing rings and PDCS all possess stable physical structures, while chaotic states are strongly random. Neural networks are more likely to learn patterns and discover potential connections between them in time series. Therefore, neural networks are more likely to capture the dynamics of single solitons, double solitons, and Turing rings, but have difficulty capturing chaotic states. We hope this method becomes a useful modeling approach for Kerr nonlinear ring resonators in the future, facilitating the control of temporal CSs and providing more possibilities for future all-optical systems and optical memory.

## Data Availability

The data that support the findings of this study are available from the corresponding author, upon reasonable request.
